# Open Circuit Fault Detection of T-Type Grid Connected Inverters Using Fast S Transform and Random Forest

**DOI:** 10.3390/e25050778

**Published:** 2023-05-10

**Authors:** Li You, Zaixun Ling, Yibo Cui, Wanli Cai, Shunfan He

**Affiliations:** 1 State Grid Hubei Electric Power Research Institute, Wuhan 430077, China; youli1987@foxmail.com (L.Y.); lingzaixun@163.com (Z.L.); pt-5@163.com (Y.C.); yanlang0304@126.com (W.C.); 2 Department of Automation, South-Central University for Nationalities, Wuhan 430070, China

**Keywords:** open-circuit fault detection, T-type inverter, fast S transform, random forest, smart grid

## Abstract

To detect open circuit faults of grid-connected T-type inverters, this paper proposed a real-time method based on fast S transform and random forest. The three-phase fault currents of the inverter were used as the inputs of the new method and no additional sensors were needed. Some fault current harmonics and direct current components were selected as the fault features. Then, fast S transform was used to extract the features of fault currents, and random forest was used to recognize the features and the fault type, as well as locate the faulted switches. The simulation and experiments showed that the new method could detect open-circuit faults with low computation complexity and the detection accuracy was 100%. The real-time and accurate open circuit fault detection method was proven effective for grid-connected T-type inverter monitoring.

## 1. Introduction

Inverters are a common and necessary interface for new types of energies and energy storage units for the grid [1,2]. Additionally, with the increasing types of energy and more intelligent control strategies, the power grid is becoming smarter than ever before [3,4]. An inverter is generally composed of switches, LC filters, and a direct current power source. The switches are most likely to be faulted because of inappropriate operation or unqualified manufacture. There are two typical faults of switches, i.e., short-circuit fault, and open-circuit fault. The short circuit is quickly detected by the protection unit and cut out. Normally, the inverter with a short-circuit fault will be stopped immediately. However, the open-circuit fault is not responded to by the protection unit and the inverter keeps running with harmonics and direct current (DC) components, which are harmful to the power grid [5,6]. Hence, detecting the open-circuit fault of grid-connected inverters is essential for the health of the smart grids.

Three-level inverters are often used in high-voltage-level or large power grids. There are two main types of three-level inverters, i.e., neutral-point clamped inverters and T-type inverters. Both of these inverters have 12 switches, but share different circuit topologies [7]. The T-type inverters have no clamping diodes and no requirement for large flying capacitors, which reduces the power consumption and risk of capacitor fault of the inverter. There are three main kinds of methods for the open-circuit fault detection of inverters: data-driven methods, model-based methods, and signal processing methods.

Data-driven methods use the fault signal directly to train a model and use it to predict the open circuit fault [8]. The deep learning method was used in [9], a neural network was used in [10], and a relevance vector machine was used in [11]. The advantages of these methods are that they require no human involvement and they possess quick fault detection. However, the disadvantages are that the trained model is highly dependent on big training data and is not flexible with grid disturbances.

Model-based methods detect open-circuit faults based on the modeling of the physical inverter systems. Sub-space state variables are employed to create an inverter system, and the changing values of the state variables indicate different types of faults [12,13]. The advantages of these methods are their clear fault detection mechanisms and explainable results, while reliance on system parameters such as resistances and inductions of the power grid impair their further application in grids with insufficient knowledge of these parameters.

Signal processing methods find fault features by fault mechanism analysis and use signal processing methods to extract features such as wavelet transform [14], *dq* transform [15], fold lines [16], and Kalman filter [17]. Then, the extracted features of the signals are recognized by manual thresholds or machine learning to detect the faults. Signal processing methods take advantages of model-based methods, exploiting the fault mechanism, and of data-driven methods, detecting the faults by artificial intelligence.

In order to detect open-circuit faults without additional sensors, inverter DC voltage and currents are often used. By the analysis of the fault mechanism and faulted current waveforms, it is found that the time-varying DC components and harmonics can reflect the open-circuit fault of the T-type inverter. Hence, time–frequency methods can be useful in fault detection. Fast S transform is a time–frequency analysis method with low computation complexity and tunable resolution which can extract time-varying fault features accurately and quickly. Random forest is a classical, accurate, and few-parameter machine-learning method that can be used to train fault detection models. Then, the model is used to monitor the fault status of the inverter.

The merits of this work and the techniques used herein are as follows:

(1) The open-circuit fault of a T-type inverter can be detected with high accuracy even if there are load changes;
(2) The fault detection uses fast S transform and random forest, which can accommodate real-time applications;
(3) The fault detection requires no manual threshold and additional sensors, which make the method useful in real industrial applications.

This paper is organized as follows: Section 2 shows the design of the new method; Section 3 gives the simulation and comparison of the new methods with existing methods; Section 4 shows the effectiveness of the experiments; and Section 5 summarizes the whole work.

## 2. The Proposed Method

To extract the open-circuit fault features of the T-type inverter, the fault mechanism should be studied first.

### 2.1. Fault Mechanism Analysis

**Figure 1:**
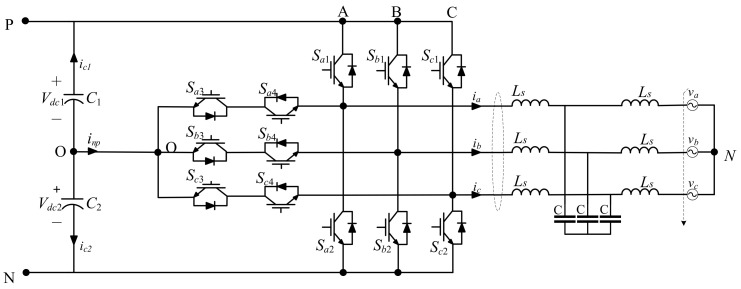
The T-type grid-connected inverter system.

**Figure 2:**
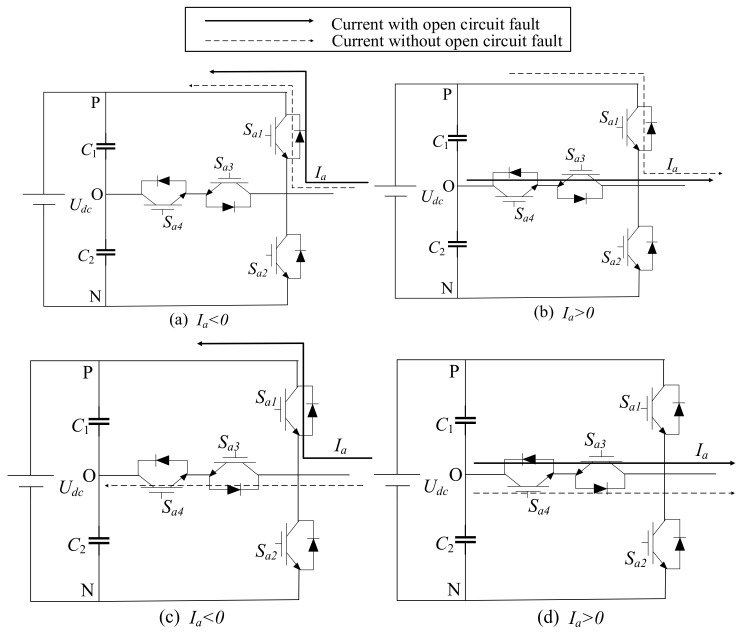
The current path of the T-type grid-connected inverter open-circuit fault.

The T-type grid-connected inverter system is shown in Figure 1. By space vector pulse width modulation (SVPWM), the 12 switches $S_{\Phi i}$ (Φ = A, B, or C, *i* = 1, 2, 3, 4) generate a three-phase alterative current (AC) to the power grid. The $S_{\Phi 1}$ and $S_{\Phi 2}$ are symmetrical, and $S_{\Phi 3}$ and $S_{\Phi 4}$ are symmetrical. Taking phase A, for instance, the current with $S_{a1}$ open-circuit fault and the one without the fault are compared in Figure 2a,b, and the current with $S_{a3}$ open-circuit fault and the one without the fault are compared in Figure 2c,d, respectively.

**Figure 3:**
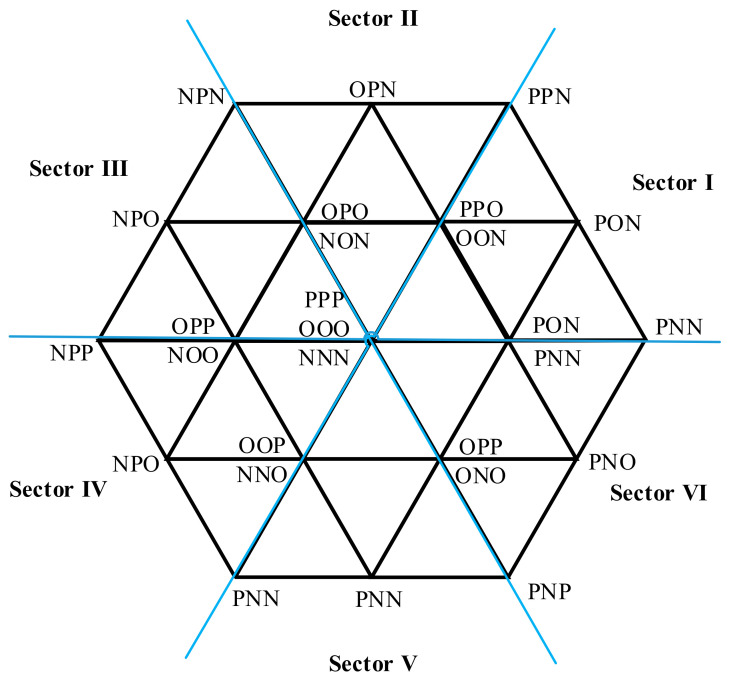
Space vector diagram of T type inverter operation.

The T-type inverter works with space vector pulse width modulation (SVPWM), which is shown in Figure 3. The space is divided into six sectors and each sector has four areas. According to the amplitude of the voltage vectors, it can be classified as zero vector U0(PPP, OOO, NNN), small-positive vector USP(POO, PPO, OPO, OOP, OPP, POP), small-negative vector USM(NOO, NNO, ONO, OON, ONN, NON), middle vector UM(PON, OPN, NPO, NOP, ONP, PNO), and large-vector UL(PNN, PPN, PNP, NPN, NPP, NNP). The phase current can be written as follows:

(1) $$i_{\Phi }=u_{\Phi }/Z_{\Phi }$$

The $u_{\Phi }$ is related to the status of the voltage vector, and when there is an open-circuit switch, the status of the voltage vector is changed, and the $i_{\Phi }$ is changed correspondingly. Taking phase A, for instance, the current with $S_{a1}$ open-circuit fault and the one without the fault are compared in Figure 2a,b, and the current with $S_{a3}$ open-circuit fault and the one without the fault are compared in Figure 2c,d respectively.

In Figure 2a,b, the $S_{a1}$ open-circuit fault turns the output state P to O when the *I_a_* is larger than 0. Then the positive *I_a_* will decrease. In Figure 2c,d, the $S_{a3}$ open circuit fault turns the output state O to P when the *I_a_* is smaller than 0. Then, the negative *I_a_* will increase. The open circuit fault of $S_{a2}$ and $S_{a4}$ are similar to the ones of $S_{a1}$ and $S_{a3}$, respectively, which are not presented here.

### 2.2. Fault Feature Analysis

**Figure 4:**
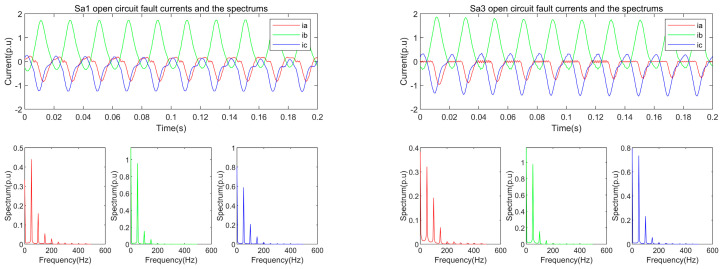
The open circuit currents and the spectrums of S_a1_ and S_a3_ respectively.

The fault currents of $S_{a1}$ and $S_{a3}$ (*I_a_*_1_ and *I_a_*_3_) are shown in Figure 4a,b respectively, and the frequency spectrums of the *I_a_*_1_ and *I_a_*_3_ are shown in Figure 4c,d respectively.

In Figure 4, it can be found that the energies of the DC component, fundamental component (first harmonic), the second harmonic, and the third harmonic contain over 95% energy of the faulted currents. Furthermore, the two fault currents have different second and third harmonics. Hence, the DC component and the two harmonic components were used as the features of the open circuit fault. Open circuit fault on different switches results in different fault currents. Taking phase A, for instance, although both $S_{a1}$ fault and $S_{a3}$ fault make the upper-half of the sinusoidal current waveform disappear, the shapes of the two corresponding distorted waveforms are different. The detailed explanation can be found in [18]. The different distorted waveforms have different spectra, which are shown in Figure 4. Our method captures the dynamic harmonic features of the fault current to detect and locate the open-circuit faulted switch.

### 2.3. Fault Feature Extraction

S transform is a powerful time–frequency analysis method that can extract the nonstationary harmonics accurately [19]. Denote the S transform of signal *x*(*t*) as

(2) $$S(\tau ,f)=\int_{-\infty }^{\infty }x(t)g(\tau -t,f)e^{-j2\pi ft}dt$$

(3) $$g(\tau -t,f)=\frac{1}{\sigma \sqrt{2\pi }}e^{0.5{\left(\frac{\tau -t}{\sigma }\right)}^{2}}$$

For a sampled current signal, the process of discrete ST can be generally divided into four steps:

(1) Use Fourier transform (FT) to the x(t) and obtain the spectrum H(m) where m is the frequency sample index (m < N);
(2) Shift H(m) with n (n < N);
(3) Compute the FT of the Gaussian window:
  (4) $$G(m,n)=\exp(-\sigma (2\pi^{2}m^{2}{/n}^{2}))$$
  where σ is the parameter to tune the shape of the Gaussian window;
(4) Multiply each H(m+n) with the corresponding G(m,n) and use Inverse FT to the result. Then, the discrete ST is obtained as
  (5) $$\left\{\begin{array}{l} S[\frac{n}{NT},jT]=\sum_{m=0}^{N-1}H(\frac{m+n}{NT})G(m,n{)e}^{i2\pi mj/N},\;\;\;\;\;\;\;\;\;\;\;\;\;\;\;\;\;n\neq 0\\ S[0,\;\;jT]=\frac{1}{N}\sum_{m=0}^{N-1}H(mT),\;\;\;\;\;\;\;\;\;\;\;\;\;\;\;\;\;\;\;\;\;\;\;\;\;\;\;\;\;\;\;\;\;\;\;\;\;\;\;\;\;\;\;\;\;\;\;\;\;\;\;\;\;n=0 \end{array}\right.$$
  where *N* is the number of signal points, *T* is the sample interval, and m,n,j∈[0,N−1]. The S[0,  jT] is the DC component of the current signal.

It should be noticed that the amplitude of the inverter current can be changed by the load. The three-phase currents *I_a_*, *I_b,_* and *I_c_* can be transformed as [20]

(6) $$\left[\begin{array}{l} I_{\alpha }\\ I_{\beta } \end{array}\right]=\frac{2}{3}\left[\begin{array}{l} 1\;-\frac{1}{2}\;-\frac{1}{2}\\ 0\;\;\;\;\frac{\sqrt{3}}{2}\;-\frac{\sqrt{3}}{2} \end{array}\right]\left[\begin{array}{l} I_{a}\\ I_{b}\\ I_{c} \end{array}\right]$$

and the load current value can be obtained by

(7) $$I_{L}=\sqrt{I_{\alpha }^{2}+I_{\beta }^{2}}$$

Denote the amplitudes of the DC component, second harmonic, and third harmonic as A_0_, A_2,_ and A_3_, respectively, then the normalized features without the load change interference can be designed as follows:

(8) $${\bar{A}}_{i}=\frac{A_{i}}{I_{L}},\;\;\;(i=0,\;\;\;\;\;2,\;\;\;\;3)$$

From the process of discrete S transform of an *N* sample signal, it can be seen that the computation complexity is *O*(*N*^3^). In fact, only half of *H* and *G* are needed because of the redundancy of FT, and the computation complexity is reduced to *O*(0.5*N*^3^). Step 1 to Step 3 are the FT of the input signal and Gaussian window, which can be realized by Fast FT (FFT). Step 4 is the inverse FT of the product of H and G, which can be realized by inverse FFT. Then, the computation complexity of the fast S transform is *O*(0.5*N*^2^log *N*), which is much lower than the one of the original S transform.

### 2.4. Random Forest for Fault Detection

The features are different with different open-circuit faults. Hence, the feature recognition needs a nonlinear classifier. Random forest has few parameter settings, low computation complexity, and a low risk of overfitting, and is useful for recognizing the fault features.

A random forest is an ensemble of decision trees [21]. Many weak classifiers make a strong one based on a voting strategy. The steps of random forest are as follows:

(1) Using bootstrap resampling on data set ***D*** to obtain a training set ***S*** = {(***F****_i_*, *L_i_*), *i* = 1,2,…,*n*}, where, ***F****_i_*, *L_i_* are the feature set and label of the *i*-th sample, respectively. The ***F*** is a set of *M*-rated harmonic amplitudes;
(2) Constructing classification and regression trees based on the ***S*** with $\sqrt{M}$ features, randomly selecting from ***F***. CART uses the Gini index (*GI*) to split the tree.
  (9) $$GI=1-\sum_{c}^{C}p(\frac{c}{s})$$
  where $p(\frac{c}{s})=\frac{n_{c}(s)}{n(s)},\;\;\sum_{c=1}^{C}p^{2}(\frac{c}{s})=1$, and $p(\frac{c}{s})$ is the probability that *s* belongs to *c*, n(s) is the number of samples in the training set whose value is *s*, $n_{c}(s)$ is the number of samples in the training set which belong to *c*, and *C* is the number of classes. The CART splits when the *GI* is minimized. Traditionally, the CART should be pruned manually, but the pruning process can be automatically carried out by the assembly learning of random forest.
(3) Repeat (1) until the tree grows to the maximum and the random forest is obtained.

When the test data are input, each tree of the random forest will return a label of the data, and the majority of the outputted labels will be the final class of the data. The T-type inverter has three phases, and each phase has three features (${\bar{A}}_{i}\;\;(i=0,\;2,\;3)$). A total of nine features are used to train the random forest, which is used to detect and located faulty switches.

## 3. Simulations

Based on the scheme of T-type grid-connected inverter system shown in Figure 1, a simulation was implemented on Matlab/Simulink. The sampling frequency was 10 kHz. The solver is a variable step with ODE45. The fault recognition algorithm was realized in Matlab. The personal computer had a Pentium i7 CPU and 16 GB of RAM. In this application, the random forest had 500 trees, and the M was 9. The σ of the S transform Gaussian window can be tuned small to obtain a quick response of the harmonic changes. Here, the σ was set as 0.1.

Because S_a1_ and S_a2_ share similar features except for the polarity of the DC component, and S_a3_ and S_a4_ shared the same similarity except for the polarity of the DC component, and only the S_a1_ and S_a3_ faults were tested in this section.

### 3.1. S_a1_ Open Circuit Fault Detection

**Figure 5:**
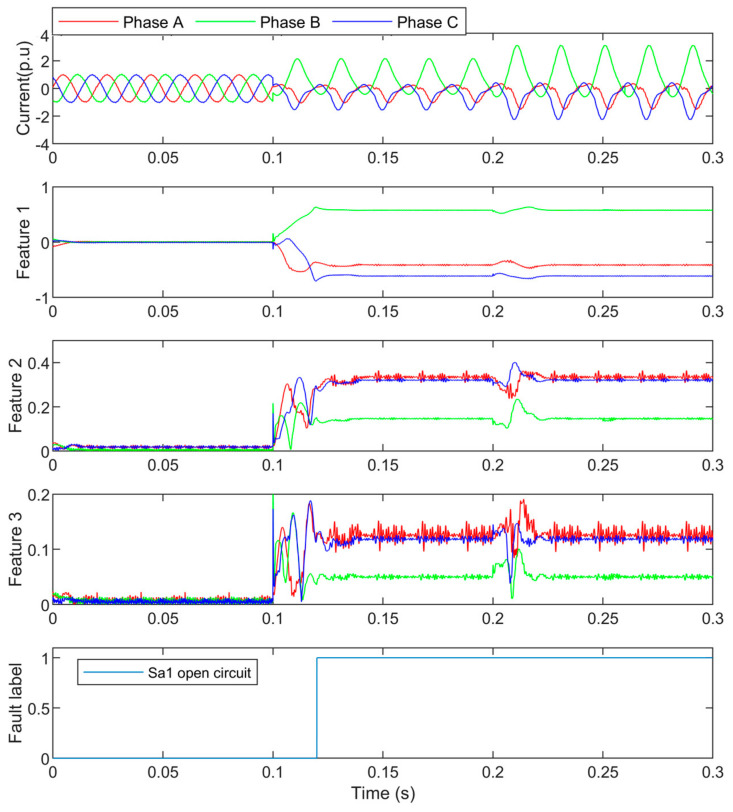
The S_a1_ open circuit currents and their detection.

In this case, S_a1_ was open-circuited at 0.1 s, and the load changed to 1.5 p.u at 0.2 s. The three-phase currents, features, and the detection result are shown in Figure 5. The feature values are presented in Table 1.

In Figure 5, it can be seen that the phase A current and phase C current have similar features except that the DC component of phase A current was about 0.5 p.u and smaller than the one of the phase C current. This is because the DC component in phase A is caused by the open circuit fault, and half of the AC is turned to half the DC waveform. While the one in Phase C is to keep *i_a_* + *i_b_* + *i_c_* = 0.

At 0.2 s the load changed to 1.5 p.u, but the features were barely changed after about one cycle transient, because the feature was normalized by the load level in Equation (7). The load changes can hardly affect the stability of the features. By the random forest process, the values of features indicate that the open circuit was in phase A, and the negative polarity of the DC component shows that the fault was with the upper arm. Then the open-circuit fault is detected and the faulted switch S_a1_ is located at about 0.12 s. The one-cycle delay is the time when the features become stable after the fault, and the random forest recognized the fault label quickly.

### 3.2. S_a3_ Open Circuit Fault Detection

**Figure 6:**
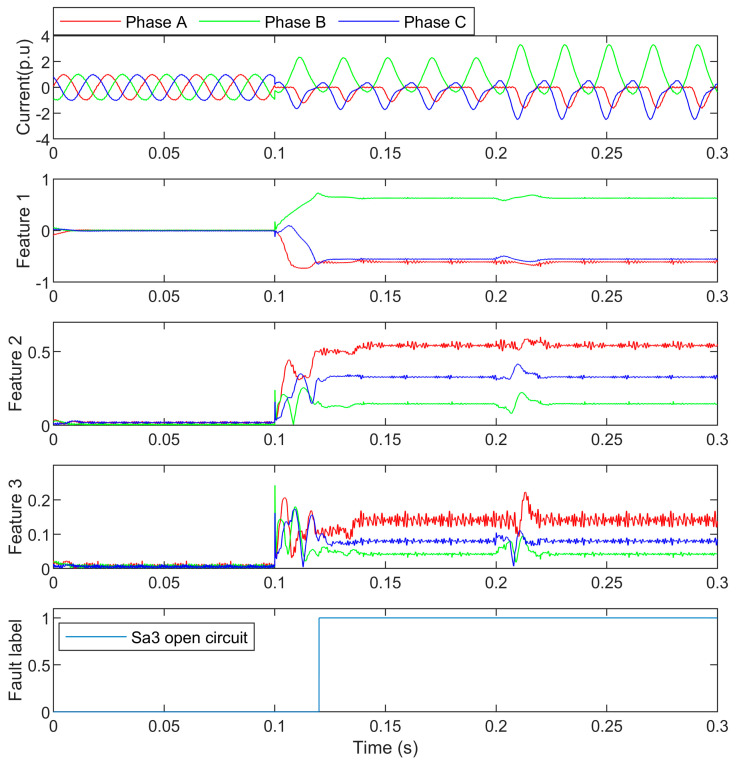
The S_a3_ open-circuit currents and their detection.

In this case, S_a3_ is open-circuited at 0.1s, and the load changed to 1.5 p.u at 0.2 s. The three-phase currents, features, and the detection result are shown in Figure 6. The feature values are presented in Table 2.

In Figure 6, it can be seen that the phase A current and phase C current had similar harmonics but smaller DC components. Additionally, compared with the features in S_a1_ open-circuit fault, the features are different in the two cases, which comply with the fault mechanisms.

At 0.2 s, the load changed to 1.5 p.u, and the features also kept stable against the load change. By the random forest process, the values of features indicate that the open circuit was in phase A, and the negative polarity of the DC component shows that the fault was with the upper arm. Then the open circuit fault was detected and the faulty switch S_a3_ was located.

## 4. Experiments

**Figure 7:**
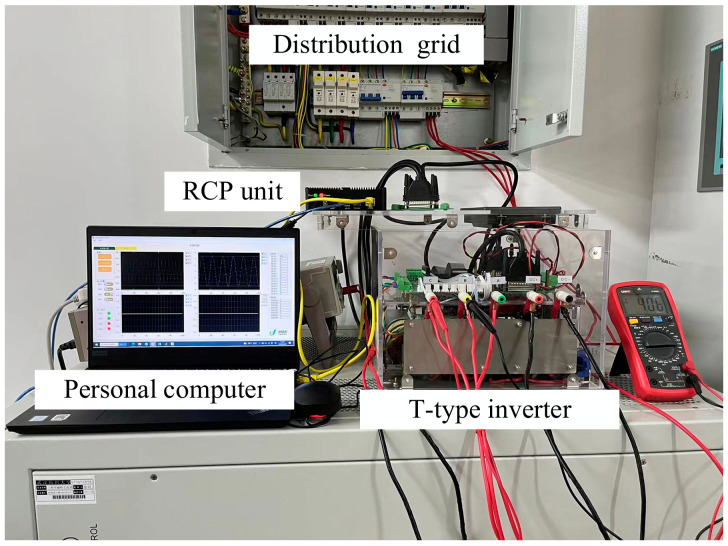
The experiment platform of the new method.

In this section, experiments of real fault detection for the grid-connected T-type inverter are shown and interpreted. The inverter was connected to a distribution grid, and the tests also featured load changes. The system included a T-type three-phase inverter, an 800 V DC power source as the DC supply, a rapid control prototyping (RCP) unit, and a personal computer. The inverter was controlled by the unit. The inverter program was realized and run by Simulink on a personal computer. The computer had an Intel i7 CPU (8 cores, 4.9 GHz) and 16 GB of RAM, and ran on a Windows 10 system, which can detect the fault in real time easily. The inverter filter inductance was 5 mH and the capacitance was 50 uF. The unit can translate the Simulink program into C code and transmit the code to the inverter. Additionally, the unit can transfer the sampled voltage and current signals of the inverter back to the computerm which uses the new method to detect and locate the open-circuit fault of the switch. The platform and inverter are shown in Figure 7.

### 4.1. S_a2_ Open Circuit Fault Detection

**Figure 8:**
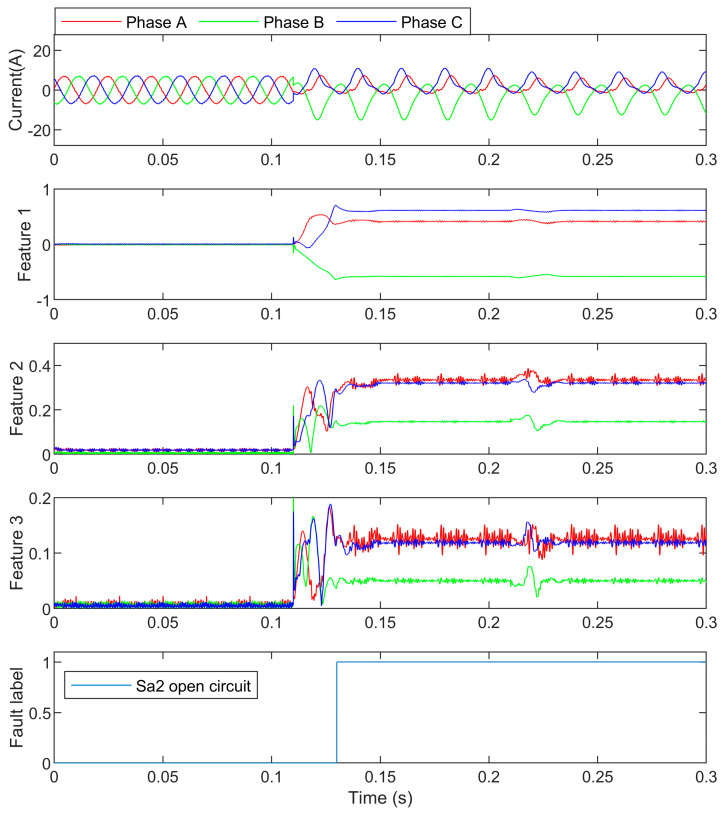
The S_a2_ open-circuit currents and their detection.

In this case, S_a2_ is open-circuited at 0.1 s, and the load changed from 7 A to 5 A at 0.2 s. The three-phase currents, features, and the detection result are shown in Figure 8. The feature values are presented in Table 3.

In Figure 8, it can be seen that the features were similar to the ones of the S_a1_ open-circuit fault except for the polarity of the DC component. It is proven that the symmetry switches have symmetrical features. The only difference is the sign of the remained half waveform. In this case, the load changed to a smaller value, but the features were still hardly changed, which shows that Equation (7) depressed the load change effect on the detection significantly.

### 4.2. S_a4_ Open Circuit Fault Detection

**Figure 9:**
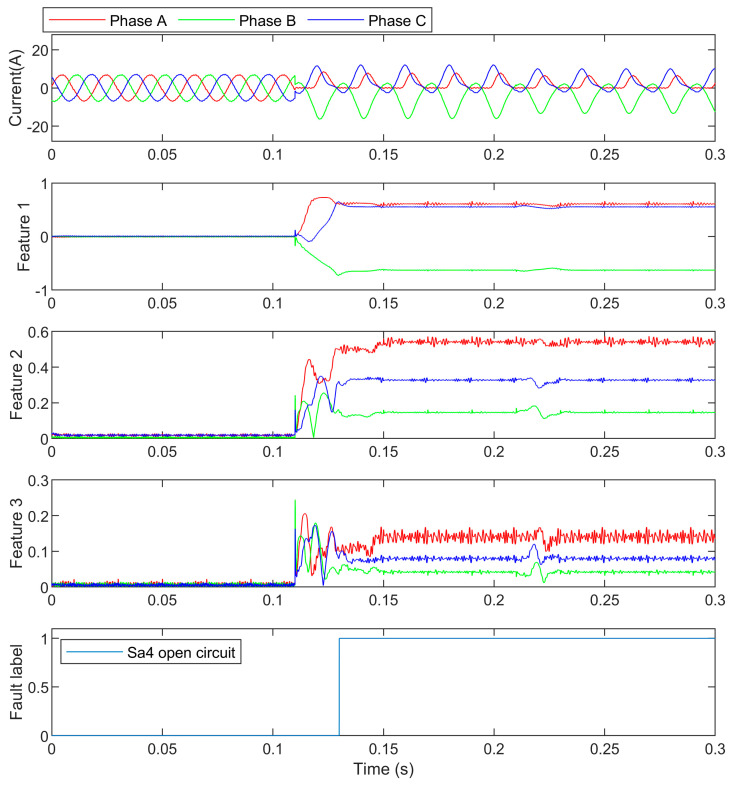
The S_a4_ open-circuit currents and their detection.

In this case, S_a4_ was open-circuited at 0.1 s, and the load changed from 7 A to 5 A at 0.2 s. The three-phase currents, features, and the detection result are shown in Figure 9. The feature values are presented in Table 4.

Compared with the S_a2_ open circuit fault, the features of this case are only different in the polarity of the DC component. The reason is that the fault waveforms share similar harmonics because of the fault mechanism but opposite the sign of waveforms. It can be seen that the load changes had little influence on the features, and can also be known that the harmonics in the faulty phase were quite different from the ones in the unfaulty phases, and random forest could recognize the fault easily.

## 5. Conclusions

This paper proposed a grid-connected T-type inverter open-circuit fault detection and locator. The fault mechanism was analyzed and the features, only based on inverter currents, were designed to reflect the mechanisms. The features were extracted by fast S transform. By normalization, load changes hardly affected the fault current features. Then, random forest was employed to recognize the features and provide a label of the faulty switches. Simulations and experiments of real faults showed that the method could detect and locate the faulty switch correctly within one cycle. The fast and accurate detection and location of the faulty switch make the new method very suitable for T-type inverter monitoring in real applications.

## Figures and Tables

**Table 1:** S_a1_ open circuit feature values.

Feature Value	Phase A	Phase B	Phase C
DC component	−0.41	0.57	−0.61
2nd harmonic	0.32	0.144	0.32
3rd harmonic	0.12	0.05	0.12

**Table 2:** S_a3_ open-circuit feature values.

Feature Value	Phase A	Phase B	Phase C
DC component	−0.55	0.63	−0.55
Second harmonic	0.53	0.15	0.32
Third harmonic	0.13	0.07	0.04

**Table 3:** S_a2_ open-circuit feature values.

Feature Value	Phase A	Phase B	Phase C
DC component	0.41	−0.57	0.60
Second harmonic	0.31	0.144	0.32
Third harmonic	0.12	0.049	0.12

**Table 4:** S_a4_ open-circuit feature values.

Feature Value	Phase A	Phase B	Phase C
DC component	0.55	−0.63	0.54
2nd harmonic	0.54	0.15	0.32
3rd harmonic	0.13	0.07	0.05

## Data Availability

Not applicable.
